# Genomic Characterization of Mobile Genetic Elements Associated with Multidrug-Resistant *Acinetobacter* Non-*baumannii* Species from Southern Thailand

**DOI:** 10.3390/antibiotics13020149

**Published:** 2024-02-02

**Authors:** Thunchanok Yaikhan, Arnon Chukamnerd, Kamonnut Singkhamanan, Natakorn Nokchan, Nutwadee Chintakovid, Sarunyou Chusri, Rattanaruji Pomwised, Monwadee Wonglapsuwan, Komwit Surachat

**Affiliations:** 1 Department of Biomedical Sciences and Biomedical Engineering, Faculty of Medicine, Prince of Songkla University, Songkhla 90110, Thailand; ythuncha@medicine.psu.ac.th (T.Y.); skamonnu@medicine.psu.ac.th (K.S.); natakorn.n@psu.ac.th (N.N.); nutwadee.c@psu.ac.th (N.C.); 2 Division of Infectious Diseases, Department of Internal Medicine, Faculty of Medicine, Prince of Songkla University, Songkhla 90110, Thailand; carnon@medicine.psu.ac.th (A.C.); sarunyouchusri@hotmail.com (S.C.); 3 Division of Biological Science, Faculty of Science, Prince of Songkla University, Songkhla 90110, Thailand; rattanaruji.p@psu.ac.th (R.P.); monwadee.wo@psu.ac.th (M.W.); 4 Translational Medicine Research Center, Faculty of Medicine, Prince of Songkla University, Songkhla 90110, Thailand

**Keywords:** *Acinetobacter* non-*baumannii*, antimicrobial resistance genes, mobile genetic elements, bacterial virulence, MDR dissemination

## Abstract

This study investigated the genetic diversity, antimicrobial resistance profiles, and virulence characteristics of *Acinetobacter* non-*baumannii* isolates obtained from four hospitals in southern Thailand. Clinical data, genome information, and average nucleotide identity (ANI) were analyzed for eight isolates, revealing diverse genetic profiles and novel sequence types (STs). Minimum spanning tree analysis indicated potential clonal spread of certain STs across different geographic regions. Antimicrobial resistance genes (ARGs) were detected in all isolates, with a high prevalence of genes conferring resistance to carbapenems, highlighting the challenge of antimicrobial resistance in *Acinetobacter* spp. infections. Mobile genetic elements (MGEs) carrying ARGs were also identified, emphasizing the role of horizontal gene transfer in spreading resistance. Evaluation of virulence-associated genes revealed a diverse range of virulence factors, including those related to biofilm formation and antibiotic resistance. However, no direct correlation was found between virulence-associated genes in *Acinetobacter* spp. and specific clinical outcomes, such as infection severity or patient mortality. This complexity suggests that factors beyond gene presence may influence disease progression and outcomes. This study emphasizes the importance of continued surveillance and molecular epidemiological studies to combat the spread of multidrug-resistant (MDR) *Acinetobacter* non-*baumannii* strains. The findings provide valuable insights into the epidemiology and genetic characteristics of this bacteria in southern Thailand, with implications for infection control and antimicrobial management efforts.

## 1. Introduction

The genus *Acinetobacter* comprises Gram-negative coccobacilli, non-fermenting, aerobic, and encapsulated bacteria commonly inhabiting soil and water. Their resilience on both moist and dry surfaces, along with resistance to common disinfectants, enables some *Acinetobacter* species to persist in hospital environments [1,2]. *Acinetobacter* non-*baumannii* is notably concerning, associated with heightened mortality, and established as a high-priority nosocomial pathogen, particularly in intensive care units (ICUs). Apart from *Acinetobacter baumannii*, the two most concerning species are *Acinetobacter pittii* and *Acinetobacter nosocomialis*, which are categorized as part of the *Acinetobacter calcoaceticus-baumannii* (ACB) complex. These species are crucial in nosocomial infections and pose a significant threat due to their resistance to multiple antibiotics and ability to thrive in healthcare environments [3]. Notably, 41% of ICU patients exhibited fecal colonization by multidrug-resistant *Acinetobacter* strains [4]. The SENTRY Program’s analysis of antimicrobial agent activity against the *Acinetobacter* spp. from 1997 to 2016 reveals a worrisome increase in multidrug-resistant (MDR) non-fermentative Gram-negative bacteria (GNB). Focusing on ACB complex isolates, 70.6% were multidrug-resistant, with 0.9% exhibiting pan-drug resistance. Colistin remains crucial, demonstrating efficacy in approximately 97% of cases [5].

Currently, many species have been included in *Acinetobacter* non-*baumannii*. The challenge lies in distinguishing these species as they share similar phenotypes and biochemical properties [6]. Accurate identification requires molecular techniques, especially sequencing, to thoroughly investigate their genomes. Sequencing emphasizes the clinical significance of *Acinetobacter* spp., which exhibits broad-spectrum antibiotic resistance, including against β-lactams, aminoglycosides, and fluoroquinolones, posing a significant treatment challenge [7]. Of particular concern are extended-spectrum β-lactamases targeting cephalosporins and carbapenems [8]. The distribution of MDR strains occurs clonally, which limits therapeutic options and reduces susceptibility to colistin. MDR isolates of *Acinetobacter* spp. have become critical nosocomial pathogens in ICU patients, being associated with ventilator-associated pneumonia, urinary tract infections, and other disorders [9]. Previous investigations highlight the distribution of the genus across various hospital wards, particularly in the ICU, emphasizing its role as a prominent source of nosocomial infections [10].

This trend may be attributed to the frequent presence of antibiotic resistance genes (ARGs) on plasmids within *Acinetobacter* strains, with potential horizontal gene transfer (HGT) to other pathogenic bacteria [11]. While global studies have explored *Acinetobacter* species outbreaks, variations in medical environments, drug regimens, and disinfection strategies contribute to diverse selective pressures. Limited research into the *Acinetobacter* non-*baumanni*i prompts our study, focusing on molecular epidemiological characteristics, ARGs, mobile genetic elements (MGEs), and sequence types (STs) in isolates from southern Thailand. This analysis aims to elucidate genetic variations, offering insights for infection treatment and control. Emphasizing the ICU as a consistent hotspot for nosocomial *Acinetobacter* spp. infections, our findings stress the need for regular disinfection protocols targeting both surfaces and air within the ICU setting.

## 2. Methodology

### 2.1. Sample Collection and Antimicrobial Susceptibility Testing

A total of 241 suspected *Acinetobacter* spp. was obtained in May 2018 as part of a short-term surveillance program conducted across four hospitals in the southern region of Thailand (Phatthalung Hospital, Satun Hospital, Pattani Hospital, and Yala Hospital). Most of the isolates were identified as *A. baumannii*, while eight were identified as *Acinetobacter* non-*baumannii*. Therefore, we selected all non-*baumannii* isolates for this study. The clinical information of the selected eight isolates is presented in Table S1. All isolates were initially identified in each hospital using conventional biochemical methods according to Bergey’s Manual of Systematic Bacteriology [12] and confirmed by matrix-assisted laser desorption/ionization time-of-flight (MALDI-TOF) mass spectrometry (MS). All isolates underwent antimicrobial susceptibility testing (AST) using the disk diffusion method, as previously described [13]. The antimicrobial disks used in this study were ciprofloxacin (5 µg), levofloxacin (5 µg), amikacin (10 µg), gentamicin (10 µg), imipenem (10 µg), meropenem (10 µg), piperacillin/tazobactam (100/10 µg), ceftazidime (30 µg), co-trimoxazole (1.25/23.75 µg), and tigecycline (15 µg). Meanwhile, the susceptibility to colistin and tigecycline was evaluated by broth microdilution method. The AST results were interpreted according to the Clinical and Laboratory Standards Institute (CLSI) standard [14], except for tigecycline. For the interpretation of tigecycline, MIC ≤ 2 μg/mL and MIC > 2 μg/mL were considered susceptible and resistant, respectively [15,16]. *Escherichia coli* ATCC^®^ 25922 (for co-trimoxazole) and *Pseudomonas aeruginosa* ATCC^®^ 27,853 were used as a quality control.

### 2.2. DNA Extraction and Sequencing

Genomic DNA from all *Acinetobacter* non-*baumanii* isolates was extracted using the QIAamp DNA Mini Kit (QIAamp Mini) (QIAGEN, Valencia, CA, USA) following the manufacturer’s guidelines. The DNA concentrations were determined using a NanoDrop™ 2000/2000c Spectrophotometer, and the integrity and purity of the DNA were confirmed by agarose gel electrophoresis. Following this, the DNA samples were submitted to the Beijing Genomics Institute (BGI) for short-read whole-genome sequencing (WGS) with 150 bp-paired end reads on the MGISEQ-2000 platform.

### 2.3. Bioinformatics and Sequence Analysis

The BacSeq pipeline [17], a bioinformatic tool designed for analyzing bacterial genomes, was used for de novo assembly, quality assessment, genome annotation, and evaluation of genome completeness. Antimicrobial resistance genes were identified using ABRicate version 1.0.1 [18] against the comprehensive antibiotic resistance database (CARD) [19]. Mobile genetic elements (MGEs) and virulence-associated genes were screened using MobileElementFinder (MEF) [20] and the virulence factor database (VFDB) [21], respectively. Additionally, the plasmid detected in this study was compared for sequence similarity to other closely related plasmids, and circular maps were visualized using Proksee version 1.0.0a6 [22].

### 2.4. Multilocus Sequence Typing and Phylogenetic Analysis

The assembly sequences of the *Acinetobacter* non-*baumannii* isolates were subjected to sequence typing (ST) analysis using the MLST method [23]. Novel STs identified in this study were submitted to the pubMLST database using Pasteur scheme [24] to contribute to the ongoing characterization of the genetic diversity of the isolates. Furthermore, ST data for *Acinetobacter* isolates from both Thailand and Malaysia were retrieved from the database to facilitate a comparative analysis of their clonal distribution. To visualize the relationships between different STs and assess the genetic relatedness of the isolates, a minimum spanning tree (MST) analysis based on the MLST profile data was constructed. The MST was generated graphically using the PHYLOViZ 2.0 program [25].

## 3. Results and Discussions

### 3.1. Clinical Data and Genome Information and Antimicrobial Susceptibility Testing Results of Acinetobacter Non-baumannii Isolates

Clinical data and genome information are exhibited in Table 1. The study presented herein involved the analysis of eight *Acinetobacter* non-*baumannii* isolates, comprising two isolates of *A. pittii*, one isolate of *Acinetobacter schindleri*, two isolates of *Acinetobacter baylyi*, and three isolates of *A. nosocomialis*. These isolates were obtained from various patient specimens across four hospitals in the southern region of Thailand, including Phatthalung Hospital, Satun Hospital, Pattani Hospital, and Yala Hospital. It is important to note that our collection efforts were confined to a specific timeframe as part of a short-term surveillance initiative. This timeframe restriction accounts for the observed limited number of isolates in our study.

**Figure 1:**
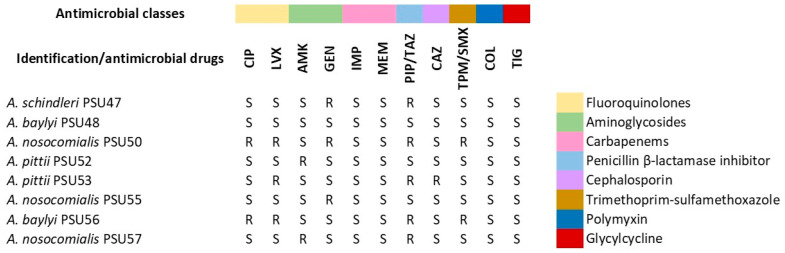
Antimicrobial susceptibility testing (AST) results of eight *Acinetobacter* non-*baumannii* isolates. Qualitative AST results were evaluated by disk diffusion, and interpretation was performed according to the CLSI standard. Abbreviations used: CIP—ciprofloxacin; LVX—levofloxacin; AMK—amikacin; GEN—gentamicin; IMP—imipenem; MEM—meropenem; PIP/TAZ—piperacillin/tazobactam; CAZ—ceftazidime; TPM/SMX—co-trimoxazole; COL—colistin; TIG— tigecycline. Interpretations: S—susceptible; R—resistant.

Despite the limited sample size, our analysis revealed valuable insights into the genomic characteristics of these isolates. The genome sizes approximately ranged from 3.3 to 4.0 Mbp, with the smallest and largest estimated genome sizes observed in *A. schindleri* and *A. nosocomialis*, respectively. The species *A. nosocomialis* and *A. pittii* belong to the *Acinetobacter calcoaceticus*-*baumannii* (ACB) complex, a group of closely related bacteria known for their ability to cause nosocomial infections. These bacteria are significant concerns in healthcare settings due to their tendency to develop resistance to multiple antimicrobial agents. According to the antimicrobial susceptibility testing (AST) results (Figure 1), *A. nosocomialis* PSU50, *A. pittii* PSU52, and *A. pittii* PSU53 were found to be multidrug-resistant strains as they resisted antimicrobial drugs from more than three classes. Both species are recognized for their capacity to develop resistance to multiple antibiotics, often demonstrating resistance to commonly used antibiotics in clinical settings, such as carbapenems, aminoglycosides, and fluoroquinolones [26,27]. Infections caused by MDR non-ACB strains, such as *A. baylyi* and *A. schindleri*, have been documented in intensive care units and tertiary care hospitals but appear to be relatively infrequent. Among the non-ACB strains, we only detected *A. baylyi* PSU56 as an MDR strain. However, previous studies have reported the incidence of *A. schindleri* among hospitalized patients. For example, in 2001, Nemec et al. investigated 22 strains of *A. schindleri* isolated from human clinical specimens [28]. Similarly, Dortet et al. reported the prevalence of infections caused by *A. schindleri*, representing 4.8% of non-ACB cases [29]. Consequently, it is noteworthy as a pathogen causing infections that should not be underestimated. 

### 3.2. Detection of Antimicrobial Resistant Genes (ARGs) from Acinetobacter Non-baumannii

**Figure 2:**
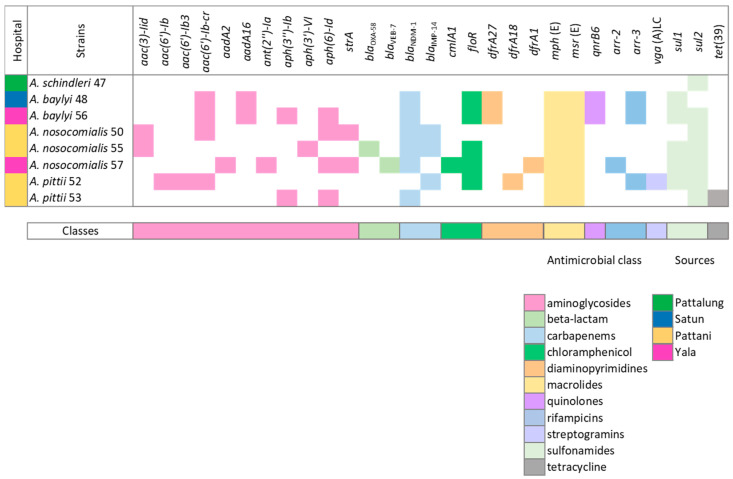
The antimicrobial resistance genes’ profile of *Acinetobacter non-baumannii*. The source of isolation is indicated by different colors on the left, while the classes of antimicrobial resistance genes are represented by colors below.

There were 29 ARGs identified in 8 *Acinetobacter* non-*baumannii* isolates, which were classified into 11 classes, including aminoglycoside, beta-lactam, carbapenems, chloramphenicols, diaminopyrimidines, macrolides, quinolones, rifampicins, streptogramins, sulfonamides, and tetracyclines. However, our results showed discrepancies between the detected ARGs and the phenotypes reported above. These discrepancies might be due to various factors, for example, the variations in antimicrobial testing, including differences in laboratory protocols, could contribute to discrepancies as we only tested for drug susceptibility according to CLSI standards. Nevertheless, the database used for ARG detection comprehensively reported all predicted ARGs that could possibly be carried by the strains. Moreover, the presence of ARGs does not always correspond to their expression or functionality, meaning that the presence or absence of a single gene may not consistently indicate whether an isolate is resistant or sensitive [30]. For the results of ARG detection in our *Acinetobacter* spp., 87.5% (7/8) of isolates were predicted as highly resistant GNB by harboring various classes of antimicrobials, including carbapenems. As shown in Figure 2, only *A. schindleri* PSU47 was detected harboring *sul2*. This concordance with previous findings suggests that this particular isolate exhibited greater susceptibility to antibiotic agents compared with both the ACB and non-ACB species [29]. However, caution is needed when interpreting these findings for the entire species, as they are based on a single isolate and may not fully represent the broader variability within the species. All isolates carrying more than two ARGs exhibited a 100% prevalence of *mph(E)* and *msr(E)*, which confers resistance to macrolides. The second highest prevalence was observed for genes conferring resistance to sulfonamides (*sul1* and *sul2*) and carbapenems (*bla*_NDM-1_). Genes encoding resistance to sulfonamides are highly prevalent among *Acinetobacter* spp. This can be elucidated by the robust association of the *sul1* gene with class 1 integrons. Meanwhile, *sul2* acquisitions are mediated by plasmids and transposons [31,32]. 

β-lactams, especially carbapenems, are in the broadest spectrum for the treatment of *Acinetobacter* spp. infections. However, increasing resistance rates have compromised their clinical utility. As in our study, seven isolates (87.5%) were detected carrying at least one metallo-β-lactamase (MBL) gene, such as *bla*_NDM-1_ and *bla*_IMP-14_. Moreover, *bla*_OXA-58_ and *bla*VEB-7 were also detected in *A. nosocomialis* PSU55 and PSU57, respectively. This is consistent with the current scenario of β-lactamases in *Acinetobacter* spp. reported by the SENTRY Antimicrobial Surveillance Program for 2020–2021, which revealed high carbapenem resistance rates in the United States and Europe. The report indicated a prevalence of *bla*_OXA_ carbapenemase genes over MBL genes, contrary to our study, which detected *bla*_NDM-1_ as the most prevalent [33]. This difference could be explained by the regional distribution and HGT of MGEs. The acquisition of ARGs in the *Acinetobacter* species has also been associated with the transfer of plasmids; therefore, isolates in the same regions could contain a similar set of ARGs.

Aminoglycosides are a class of antibiotics extensively applied in the treatment of *Acinetobacter* spp. infections. However, the clinical isolates commonly exhibit high resistance to traditional agents such as gentamicin and kanamycin [34]. The resistance mechanisms to aminoglycoside agents in *Acinetobacter* primarily involve the production of aminoglycoside-modifying enzymes, which can be categorized as aminoglycoside acetyltransferases (AAC), aminoglycoside phosphotransferases (APH), and/or aminoglycoside nucleotidyltransferases (ANT or AAD). According to the results of this study, 10 aminoglycoside-modifying genes were detected, which were *aac(3)-Iid*, *aac(6′)-Ib*, *aac(6′)-Ib3*, *aac(6′)-Ib-cr*, *aadA2*, *aadA16*, *ant(2″)-Ia*, *aph(3″)-Ib*, *aph(3′)-VI*, and *aph(6)-Id.* With the exception of *A. schindleri* PSU47, all other isolates were found to carry a minimum of two aminoglycoside resistance genes, with as many as four different genes being identified in some isolates (PSU50, 56, and 57). *Acinetobacter* spp. isolates frequently harbor these genes on mobile elements such as plasmids and transposons, facilitating their transfer within the closely related population [35].

Interestingly, *A. pittii* PSU52 was found to carry *vga*(A)_LC_. The gene is a variant of the streptogramin A resistance gene, observed in clinical isolates of *Staphylococcus haemolyticus* that are resistant to lincomycin and clindamycin [36]. There is a possibility of the transfer of resistance from Gram-positive bacteria (GPB) to GNB, particularly observed in *Staphylococcus*, through the process of transconjugation of plasmids [37]. This phenomenon will be further elaborated upon in our work, specifically in the section dedicated to the detection of MGEs. Due to the adaptive nature of *Acinetobacter* isolates, which can accumulate resistance traits through multiple mechanisms, the transfer of resistance genes from Gram-positive bacteria (GPB) to GNB can broaden the spectrum of antimicrobial resistance in a bacterial population. This enables the limitation of certain antimicrobial effectiveness in treating infections caused by bacteria that have acquired new resistance traits. Although our work showed the same species collected from the same hospital (*A. nosocomialis* PSU50 and PSU55 and *A. pittii* PSU52 and PSU53 from Yala hospital), the ARG profiles are not presented the same. It is important to note that resistance profiles depend on various factors, including the genetic diversity of the bacterial population, the effectiveness of infection control measures, and the specific antibiotic usage patterns in the hospital. Additionally, not all bacteria of the same species may acquire or share the same resistance genes, and variations can exist even within a single species. Therefore, comprehensive analysis could assist healthcare staff in monitoring and being concerned about the transmission of these pathogens in hospitals and potentially within the community.

### 3.3. Detection of Mobile Genetic Elements (MGEs)

**Figure 3:**
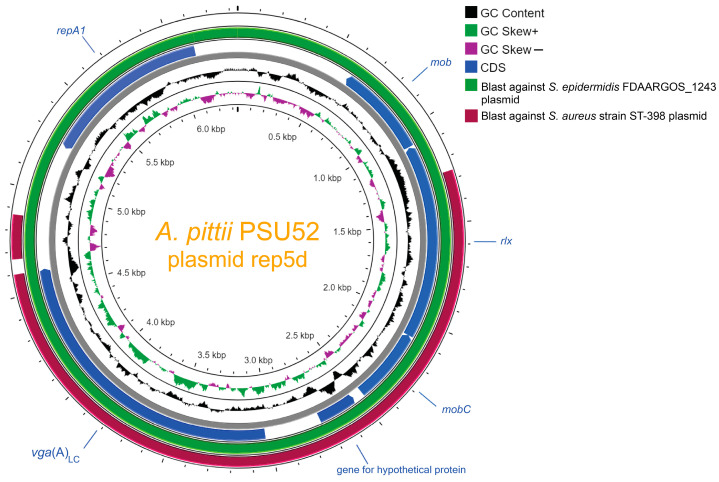
A sequence alignment was performed for the rep5d plasmid from *A. pittii* PSU52 against rep5d from Gram-positive bacteria (GPB), including *Staphylococcus epidermidis* FDAARGOS_1243 and *Staphylococcus aureus* strain ST-398. Annotated genomes of GPB were retrieved from NCBI and compared to identify conserved and divergent sequence features. The innermost circle represents GC skew (dark green for GC skew+ and magenta for GC skew−). The second circle (black) shows GC content. The dark blue color indicates the coding regions (CDs) of the rep5d plasmid from *A. pittii* PSU52 carrying the *vga*(A)_LC_ gene, with annotated genes at specific positions and arrows indicating the direction of gene transcription. The green and dark purple colors represent the CDs of the *S. epidermidis* FDAARGOS_1243 plasmid and the *S. aureus* strain ST-398 plasmid obtained from blastn similarity results, respectively. BLAST analysis, conducted using Proksee software version 1.0.0a6 on the CGView server, identified missing regions, which are represented as gaps on each of the circular genomes.

*Acinetobacter* non-*baumannii* can develop resistance to antimicrobial agents through processes such as HGT or natural transformation [38,39]. According to MobileElementFinder [20], MGEs including plasmids, insertion sequences (ISs), and composite transposons (CNs) were detected in eight isolates (Table 2). Two of these isolates were identified as carriers of ARGs located on MGEs. *A. pittii* PSU52 was found to carry *vga*(A)_LC_, a gene typically identified in GPB on a rep5d plasmid. Therefore, we further compared the rep5d plasmid from *A. pittii* PSU52 to those from *Staphylococcus epidermidis* FDAARGOS_1243 and *Staphylococcus aureus* strain ST-398 (Figure 3). They exhibited high similarity, which might indicate that the rep5d plasmid in *A. pittii* PSU52 originated from *Staphylococcus* bacteria. Typically, the transfer of conjugative plasmids from Gram-positive to Gram-negative bacteria is limited to a small number of occurrences. However, there have been reports of the transfer of antibiotic resistance genes, especially β-lactams, and carbapenems, between them previously [40,41,42]. The occupation of the *vga*(A)_LC_ gene probably does not affect the medication used to treat *Acinetobacter* infections. The first-line therapeutic options of *Acinetobacter* spp. are beta-lactam antibiotics, carbapenems, and fluoroquinolones, while second-line agents include polymyxins (such as polymyxin B and colistin) and tetracycline derivatives (for example, minocycline and tigecycline) [43]. However, it is noteworthy to be able to identify plasmids containing ARGs in completely different bacteria because it might broaden the spectrum of ARGs in a bacterial population. This can limit the effectiveness of certain antibiotics in treating infections caused by bacteria that have acquired new resistance traits. 

Carbapenems are the most important antibiotics against MDR bacterial infections, but *Acinetobacter* spp. is currently also resistant to carbapenems due to the acquisition of carbapenemases. In this study, *A. nosocomialis* PSU55 was detected carrying *bla*_IMP-14_ on cn_3572_IS1008. Previous research has also identified several types of insertion sequences associated with carbapenem resistance in the *Acinetobacter* spp. ISAba1, ISAba2, ISAba3, and IS18 are commonly linked to the expression of carbapenemase genes, with prevalence rates of 93.2%, 25.4%, 20.3%, and 5.1%, respectively [44]. However, our findings showed different prevalence rates of ISs. We identified 61 ISs, with the highest prevalence observed for IS17, ISAba33, ISAba27, IS18, and ISAha3 at 62.5%, 62.5%, 62.5%, 50%, and 50%, respectively. Differences in the prevalence rates of ISs may be caused by various factors. Genetic diversity within bacterial isolates, driven by factors such as horizontal gene transfer and antimicrobial use, can impact IS prevalence. Moreover, the possibility of emerging ISs or changes in their prevalence over time makes the situation more complicated. These factors collectively highlight the intricate nature of antimicrobial resistance in *Acinetobacter* species, underscoring the need for comprehensive studies to elucidate the molecular epidemiology of ISs and other MGEs. Therefore, a comprehensive analysis of mobilizable elements in bacteria aids in understanding the dynamics of gene transfer and tracking the prevalence of resistance genes in different bacterial populations. This information is crucial for effective surveillance and monitoring, helping healthcare providers make informed decisions about treatment strategies.

### 3.4. Evaluation of Virulence-Associated Genes in Eight Acinetobacter Non-baumannii Isolates

**Figure 4:**
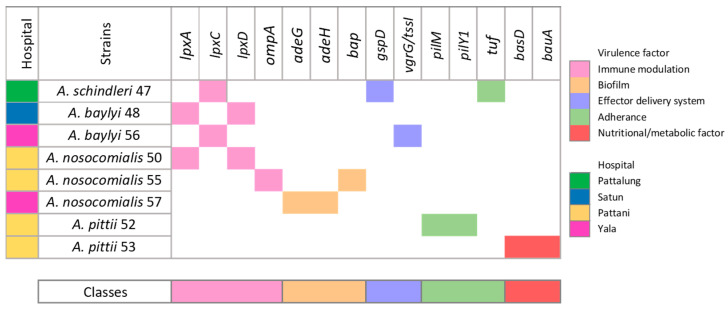
Evaluation of virulence-associated genes in *Acinetobacter* non-*baumannii*. The four hospitals of isolation are indicated by different colors and labeled on the bottom right. Classes of virulence factors are differentiated and color-coded on the top right.

*Acinetobacter* is one of the most common and significant pathogens in human infections [45]. Within this genus, *A. baumannii*, *A. pittii*, and *A. nosocomialis* are also clinically important in several regions [26]. Virulence factors are properties employed by microorganisms to induce disease within the host body. Among these factors are those that elevate *Acinetobacter* to the status of a serious pathogen, such as its capacity to tolerate high levels of stress and its increased expression of efflux pumps, resulting in high levels of antibiotic resistance [46]. In this study, we investigated and compared the virulence-associated genes of isolates from eight isolates using in silico methods. The results are shown in Figure 4. According to the virulence factor database (VFDB) [47], we identified 14 genes that belonged to 5 groups of virulence factors, including immune modulation (*lpxA*, *lpxC*, *lpxD*, *ompA*), biofilm (*adeG*, *adeH*, *bap*), effector delivery system (*gspD*, *vgrG/tssI*), adherence (*pilM*, *pilY1*, *tuf*), and nutritional/metabolic factor (*basD*, *bauA*); *lpxA*, *lpxC*, and *lpxD* are related to the inflammatory signaling pathway [47]. These genes are involved in the biosynthesis of lipid A, a component of lipopolysaccharides (LPS) in *Acinetobacter* spp. Mutations in LPS-associated genes are a concern because LPS deficiency can lead to colistin resistance in *A. baumannii*. Furthermore, Moffat et al. reported that a colistin-resistant clinical isolate had its *lpxD* gene disrupted by the insertion of an IS element [48]. They also found that ISAba11 inactivated the *lpxC* and *lpxA* genes in colistin-resistant derivatives of *A. baumannii* ATCC19606 [49]. The *ompA* gene encodes an outer membrane protein that not only facilitates cell apoptosis but also plays a role in the initial stage of biofilm formation on abiotic surfaces. Additionally, it is required for adhesion to host epithelial cells and facilitates the invasion of *Acinetobacter* spp. cells into host epithelial and immune cells [50]. Despite the identification of various common virulence genes in our study, we found no correlation between the detected virulence-associated genes and the species of the isolates or the hospital of isolation. Previous studies have reported a diverse range of virulence characteristics among *A. baumannii* isolates, indicating that their pathogenicity is not solely dependent on one virulence factor [51]. Our study also demonstrates significant diversity in the virulence genes of clinical isolates. However, it is important to note that these virulence genes are used for predicting virulence traits and may not directly correlate with clinical outcomes in patients as this study did not evaluate virulence phenotypes or the clinical characteristics of the infections. Therefore, it remains unknown whether isolates with high virulence traits in our study resulted in worse clinical outcomes for patients. The lack of direct correlation between virulence-associated genes and clinical outcomes underlines the complexity of *Acinetobacter* infections. This suggests that factors beyond genetic determinants, especially when using genome characteristics for gene prediction, may influence disease severity and treatment response in patients. Understanding these additional factors could be crucial for developing more effective therapeutic strategies customized to the specific characteristics of individual infections.

### 3.5. Multilocus Sequence Typing (MLST) and Phylogenetic Analysis

**Figure 5:**
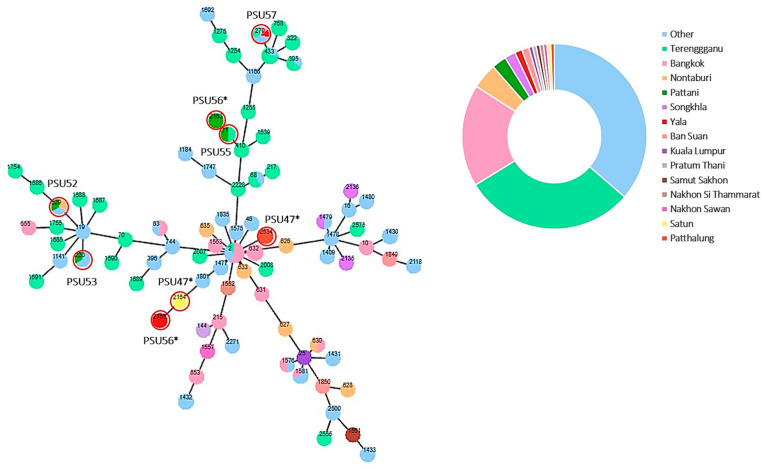
The minimum spanning tree for ST types, which was generated using *Acinetobacter* non-*baumannii* isolates obtained from Thailand and Malaysia available on the pubMLST database through the PHYLOViZ website. Each node in the tree represents a unique ST type, with the color indicating the provinces of collection. The length between two nodes reflects the genetic distance between the two bounding ST types. The strains marked with an asterisk (*) were identified as novel STs.

Eight unique sequence types (STs) were identified for the eight *Acinetobacter* non-*baumannii* isolates using pubMLST (Pasteur scheme). These included ST629, ST220, ST71, and ST279. Interestingly, four out of the eight isolates in our study were identified as novel STs as follows: ST2534, ST2163, ST2164, and ST2165 (Figure 5). Additionally, we conducted a minimum spanning tree analysis using pubMLST to investigate the potential dissemination of clones across Thailand and Malaysia. Our findings revealed that four of our study isolates shared STs with isolates from other regions: (i) *A. nosocomialis* PSU57 from Yala Hospital exhibited an identical ST to six *A. nosocomialis* isolates from Bangkok, Thailand, and Terengganu, Malaysia; (ii) *A. nosocomialis* PSU55 from Pattani Hospital, identified as ST71, shared its ST with an *A. nosocomialis* isolate from Terengganu, Malaysia; (iii) *A. pittii* PSU53 from Pattani Hospital, identified as ST220, shared its ST with two other isolates from different regions in Thailand; (iv) *A. pittii* PSU52 from Pattani Hospital was identified as ST629, which is also shared by isolates from Nonthaburi and another province in Thailand (Table S2).

The identification of novel STs among the *Acinetobacter* spp. in our study expands the genetic diversity of this pathogen. This diversity is further highlighted by the sharing of STs between isolates from different geographical regions, as seen with ST279 and ST71 from Yala and Pattani hospitals in Thailand, respectively, which shared identical STs with isolates from Bangkok, Thailand, and Terengganu, Malaysia. The dissemination of identical STs across these regions suggests potential clonal spread or common sources of these strains. The sharing of STs between isolates from different hospitals and countries raises questions about the epidemiology and transmission dynamics of *Acinetobacter* spp. in these areas. Factors such as patient movement, inter-hospital transfer, or environmental contamination could contribute to the observed spread of genetically similar strains. Furthermore, the presence of shared STs between our isolates and those from other regions underscores the importance of regional surveillance and monitoring to track the spread of specific clones. Understanding the genetic relatedness and transmission patterns of bacteria is crucial for implementing effective infection control measures and measures to optimize the use of antimicrobial agents in order to diminish the spread of MDR strains. Continued surveillance efforts, combined with detailed molecular epidemiological studies, will be essential for gaining insights into the dynamics of *Acinetobacter* non-*baumannii* transmission and for informing strategies to control its spread.

## 4. Conclusions

In this study, we investigated various aspects of *Acinetobacter* non-*baumannii* isolates obtained from four hospitals in southern Thailand. We analyzed clinical data, genome information, and ANI of the isolates. Our findings revealed diverse genetic profiles among the isolates, with differences in estimated genome sizes and ANI values. Notably, we identified novel STs and observed potential clonal spread of certain STs across different geographic regions. Additionally, we detected ARGs in the isolates, highlighting the presence of genes conferring resistance to various classes of antimicrobials. The prevalence of ARGs, especially those associated with carbapenem resistance, raises concerns about the effectiveness of antibiotics in treating *Acinetobacter* spp. infections. Our study also identified MGEs carrying ARGs, emphasizing the role of horizontal gene transfer in spreading antimicrobial resistance. Furthermore, we evaluated virulence-associated genes in the isolates and found a diverse range of virulence factors, including those related to biofilm formation and antibiotic resistance. Despite not observing a direct correlation between the presence of virulence-associated genes in *Acinetobacter* non-*baumannii* infections and specific clinical outcomes, it is important to emphasize the necessity for additional research to better understand the factors influencing disease progression and patient outcomes in these infections.

Overall, our study provides valuable insights into the genetic diversity, antimicrobial resistance profiles, and virulence characteristics of *Acinetobacter* non-*baumannii* isolates in southern Thailand. These findings have implications for infection control and antimicrobial deployment efforts, emphasizing the importance of continued surveillance and molecular epidemiological studies to combat the spread of MDR- *Acinetobacter* spp. strains.

## Figures and Tables

**Table 1:** Clinical data and genome information.

Strains	Isolation Sources	Location	Estimated Genome Size (bp) *	N_50_ Value
*A. schindleri* PSU47	Endotracheal tube	Phatthalung	3,347,770	93,667
*A. baylyi* PSU48	Nasopharynx	Satun	3,601,820	79,944
*A. nosocomialis* PSU50	Rectum	Pattani	4,015,432	122,902
*A. pittii* PSU52	Rectum	Pattani	3,986,945	110,976
*A. pittii* PSU53	Rectum	Pattani	3,917,437	75,803
*A. nosocomialis* PSU55	Endotracheal tube	Pattani	4,047,440	93,952
*A. baylyi* PSU56	Nasopharynx	Yala	3,820,703	216,777
*A. nosocomialis* PSU57	Rectum	Yala	4,091,066	103,279

* Draft genome length was obtained using short-read sequencing.

**Table 2:** Mobile genetic elements and the carried antimicrobial resistance genes.

Isolates	Mobile Genetic Elements (MGEs)			ARGs Found on MGEs
	Plasmid	Insertion Sequences (*n* **)	Composite Transposons	
*A. schindleri* PSU47		ISOur1, IS1007, ISAca1, ISAcsp5, IS18, ISAba11	cn_2205_ISOur1, cn_2205_IS1007	
*A. baylyi* PSU48		IS17(3), ISAba27, ISAba34,		
		ISAcsp5 *		
*A. nosocomialis* PSU50		ISAba26, ISAba13, IS17, ISAha3, ISAba27, ISAba33 (2), ISAba1, ISOur1, IS18, ISAba14	cn_26436_ISAba33, cn_8866_IS18	
		ISCfr1 *		
*A. pittii* PSU52	rep5d *			*vga*(A)_LC_
		ISAba45, ISAba26, ISAba50, ISAba13, IS17, ISAba33, IS26	cn_4415_ISAba33	
*A. pittii* PSU53		ISAba1, ISAba33, ISAba26, ISPst2, ISAha3, ISAba31		
*A. nosocomialis* PSU55			cn_3572_IS1008 *	*sul2*, *aac(3)-Iid*, *sul1*, *bla*_IMP-14_
		ISCfr1 *		
		IS1008, ISAba14, ISAba1, ISAba33, ISAba10(2), IS17, ISAha3, ISAba27, ISAba25		
*A. baylyi* PSU56		IS17 *		
		IS18 *		
		IS17(2), ISAba27, ISAba14		
*A. nosocomialis* PSU57		IS18, ISAba125, ISAba27(2), ISAha3, ISAba33, ISAba21, IS1007	cn_8866_IS18, cn_1062_IS1007, cn_2153_IS1007	

* MGEs that carry ARGs. ** Number in the parenthesis represents the number of detected MGEs.

## Data Availability

The genomes of all *Acinetobacter* non-*baumannii* isolates assembled in this study have been archived and are accessible through BioProject number PRJNA1055677, with corresponding BioSample numbers ranging from SAMN39059577 to SAMN39059584.

## Supplementary Materials

The following supporting information can be downloaded at: https://www.mdpi.com/article/10.3390/antibiotics13020149/s1, Table S1: Clinical information for *Acinetobacter calcoaceticus*-*baumannii* complex isolated from this study; Table S2: Demographic Data for Acinetobacter calcoaceticus-baumannii Isolates in Thailand and Malaysia Collected from the pubMLST Database.
