# Supplementary material for: Genomic Characterization of Mobile Genetic Elements Associated with Multidrug-Resistant Acinetobacter Non-baumannii Species from Southern Thailand
Source: Antibiotics (Basel). 2024 Feb 2;13(2):149. doi: 10.3390/antibiotics13020149 (PMC10886146; doi:10.3390/antibiotics13020149)
Supplement: Supplementary file 1 [file antibiotics-13-00149-s001.zip › antibiotics-2813194-supplementary.pdf]

antibiotics

MDPI

Supplementary Materials

Genomic Characterization of Mobile Genetic Elements Associated with Multidrug-Resistant *Acinetobacter* Non-*baumannii* Species from Southern Thailand

Thunchanok Yaikhan [1], Arnon Chukamnerd [2], Kamonnut Singkhamanan [1], Natakorn Nokchan [1], Nutwadee Chintakovid [1], Sarunyou Chusri [2], Rattanaruji Pomwised [3], Monwadee Wonglapsuwan [3] and Komwit Surachat [1,4,*]

[1] Department of Biomedical Sciences and Biomedical Engineering, Faculty of Medicine, Prince of Songkla University, Songkhla 90110, Thailand; ythuncha@medicine.psu.ac.th (T.Y.); skamonnu@medicine.psu.ac.th (K.S.); natakorn.n@psu.ac.th (N.N.); nutwadee.c@psu.ac.th (N.C.)
[2] Division of Infectious Diseases, Department of Internal Medicine, Faculty of Medicine, Prince of Songkla University, Songkhla 90110, Thailand; carnon@medicine.psu.ac.th (A.C.); sarunyouchusri@hotmail.com (S.C.)
[3] Division of Biological Science, Faculty of Science, Prince of Songkla University, Songkhla 90110, Thailand; rattanaruji.p@psu.ac.th (R.P.); monwadee.wo@psu.ac.th (M.W.)
[4] Translational Medicine Research Center, Faculty of Medicine, Prince of Songkla University, Songkhla 90110, Thailand
* Correspondence: komwit.s@psu.ac.th

Table S1. Clinical information for *Acinetobacter calcoaceticus-baumannii* complex isolated from this study.

No.	Hospital	Source of isolation	Ward of isolation	Identification (MALDI)
47	PT	Tu/1	ICU	*Acinetobacter schindleri*
48	ST	Ng	ICU	*Acinetobacter baylyi*
50	PA	R/2	ICU	*Acinetobacter nosocomialis*
52	PA	R/2	ICU	*Acinetobacter pittii*
53	PA	R/2	ICU	*Acinetobacter pittii*
56	YL	Ng	ICU	*Acinetobacter baylyi*
55	PA	Tu/2	ICU	*Acinetobacter nosocomialis*
57	YL	R	ICU	*Acinetobacter nosocomialis*

Hospital	Source of Isolation
PT: Patthalung Hospital	Ng: nasopharynx
ST: Satun Hospital	Tu: endotracheal tube
YL: Yala Hospital	R: rectum
PA: Pattani Hospital	

Table S2. Demographic Data for *Acinetobacter calcoaceticus-baumannii* Isolates in Thailand and Malaysia Collected from the pubMLST Database.

ST	Isolate	Country	Region	Year	Species	Source
71	AC1614	Malaysia	Terengganu	2016	*Acinetobacter nosocomialis*	wound
71	PSU55*	Thailand	Pattani	Null	*Acinetobacter nosocomialis*	endotracheal tube
220	AP864	Thailand	null	2018	*Acinetobacter pittii*	sputum
220	AP984	Thailand	null	2018	*Acinetobacter pittii*	abscess
220	PSU53*	Thailand	Pattani	2018	*Acinetobacter pittii*	nasopharynx
279	T228	Thailand	Bangkok	2010	*Acinetobacter nosocomialis*	Sputum
279	PSU57*	Thailand	Yala	2018	*Acinetobacter nosocomialis*	Rectum
279	4300STDY7045890	Thailand	null	2018	*Acinetobacter nosocomialis*	null
279	4300STDY7045734	Thailand	null	null	*Acinetobacter nosocomialis*	null
279	4300STDY7045717	Thailand	null	null	*Acinetobacter nosocomialis*	null
279	AC1572	Malaysia	Terengganu	2015	*Acinetobacter nosocomialis*	sputum
279	AC15181	Malaysia	Terengganu	2015	*Acinetobacter nosocomialis*	blood
629	DMST35109	Thailand	Nonthaburi	2011	*Acinetobacter baumannii*	null
629	4300STDY7045719	Thailand	null	null	*Acinetobacter pittii*	null
629	PSU52*	Thailand	Pattani	2018	*Acinetobacter pittii*	nasopharynx
2163	PSU50*	Thailand	Pattani	2018	*Acinetobacter nosocomialis*	null
2164	PSU48*	Thailand	Satun	2018	*Acinetobacter baylyi*	null
2165	PSU56*	Thailand	Yala	2018	*Acinetobacter baylyi*	null
2534	PSU47*	Thailand	Patthalung	2018	*Acinetobacter schindleri*	null
